# Fluorescence Tumor-Imaging Using a Thermo-Responsive Molecule with an Emissive Aminoquinoline Derivative

**DOI:** 10.3390/nano8100782

**Published:** 2018-10-02

**Authors:** Takeru Araki, Yasufumi Fuchi, Shuhei Murayama, Ryoma Shiraishi, Tokimi Oyama, Mariko Aso, Ichio Aoki, Shigeki Kobayashi, Ken-ichi Yamada, Satoru Karasawa

**Affiliations:** 1 Graduate School of Pharmaceutical Sciences, Kyushu University, 3-1-1 Maidashi, Higashi-Ku, Fukuoka 812-8582, Japan; araking417@gmail.com (T.A.); oirawawhitydaze0709@gmail.com (R.S.); aso@phar.kyushu-u.ac.jp (M.A.); kenyamada@phar.kyushu-u.ac.jp (K.Y.); 2 Faculty of Pharmaceutical Sciences, Showa Pharmaceutical University, Machida, Tokyo 194-8543, Japan; fuchi@ac.shoyaku.ac.jp (Y.F.); b14030@ug.shoyaku.ac.jp (T.O.); kobayasi@ac.shoyaku.ac.jp (S.K.); 3 Department of Bioanalytical Chemistry, School of Pharmacy, Showa University, 1-5-8 Hatanodai, Shinagawa-ku, Tokyo 142-8555, Japan; s.murayama@pharm.showa-u.ac.jp; 4 Department of Molecular Imaging and Theranostics, National Institute of Radiological Sciences (NIRS), QST, Anagawa 4-9-1, Inage, Chiba 263-8555, Japan; iaoki.jp@gmail.com; 5 PRESTO, Japan Science and Technology Agency, Kawaguchi 332-0012, Japan

**Keywords:** nanoparticles, lower critical solution temperature, fluorescence, self-assembly, tumor-imaging

## Abstract

We synthesized (2,4-trifluoromethyl-7-*N*-bis(2,5,8,11-tetraoxatridecane-13-yl)-aminoquinoline) TFMAQ-diEg4, an emissive aminoquinoline derivative that incorporated two tetraethyleneglycol chains into an amino group. TFMAQ-diEg4 showed fluorescence and thermo-responsive properties accompanied by a lower critical solution temperature (LCST), due to the introduction of the oligoethylene glycol chain. This thermo-responsive LCST behavior occurred at the border of a cloud point. Below and above the cloud point, self-assemblies of 6-7-nm nanoparticles and ~2000-nm microparticles were observed, in vitro. In addition, TFMAQ-diEg4 showed a high solubility, over 20 mM for aqueous solution, in vivo, which not only prevented thrombosis but also allowed various examinations, such as single intravenous administration and intravenous drips. Intravenous administration of TFMAQ-diEg4, to tumor-bearing, mice led to the accumulation of the molecule in the tumor tissue, as observed by fluorescence imaging. A subset of mice was treated with local heat around their tumor tissue and an intravenous drip of TFMAQ-diEg4, which led to a high intensity of TFMAQ-diEg4 emission within the tumor tissue. Therefore, we revealed that TFMAQ-diEg4 was useful as a fluorescence probe with thermo-responsive properties.

## 1. Introduction

Tumor tissues are differentiated from healthy tissues on the basis of a number of different properties. For example, three of the most well-known features are, (1) the presence of a void space in the tumors [1,2], (2) acidic properties of tumors [3,4], and (3) higher temperature of tumor tissues [5,6]. The void spaces, which form when cancer cells undergo apoptosis, are approximately 10–500 nm in size; therefore, nanoparticles of this size have been shown to penetrate and remain within tumors. This is described as the enhanced permeability and retention (EPR) effect [7] and is very useful for drug delivery systems (DDS). As such, many DDS researchers have begun to take advantage of this tumor-specific effect [8,9]. Differences in the pH of tumor tissue are due to the production of lactic acid, which causes tumors to maintain a pH of 6.5 to 7.0 (certain tumors reach an even more acidic pH of 5.0) [10], which is substantially lower than the pH of healthy tissues (~7.4). Lastly, tumor tissues exhibit higher temperatures than those of healthy tissues. For example, Hayashi et al. reported that the temperature of breast cancer tissue is 1−2 °C higher than that of healthy tissue [11]. 

**Scheme 1:**
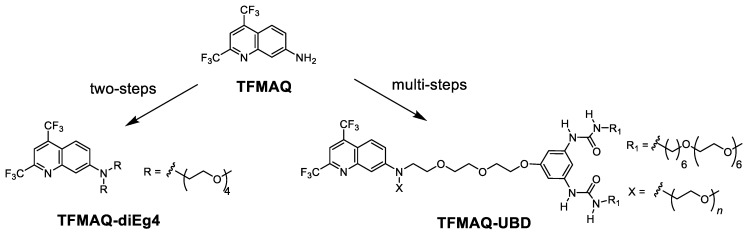
Molecular structures of TFMAQ, TFMAQ-diEg4, and TFMAQ-UBD.

On the contrary, recent studies on thermos-responsive materials, such as poly(*N*-isopropylacrylamide) [12] and poly(2-isopropyl-2-oxazoline) [13], have gathered much attention in the field of DDS. We have studied self-assembled molecules possessing thermo-responsive properties [14,15,16,17]. Ureabenzene derivatives (UBD) possess amphiphilic side chains, such as alkyl chains and oligoethylene glycol (OEG), and have been shown to have a self-assembly behavior that is triggered by heat. The self-assembly of UBD is mediated by the response of OEG to heat, wherein, heat induces dehydration [18,19] and the molecule then self-assembles by hydrophobic interactions. Therefore, we prepared a thermo-responsive fluorescent molecule TFMAQ-UBD (*n* = 3, 4, and 6, Scheme 1) [20], made up of UBD and an aminoqunoline derivative (TFMAQ) [21,22,23,24], which a fluorophore that possesses medium emission quantum yields. Accordingly, TFMAQ-UBD showed a self-assembly behavior at a low temperature, which led to the formation of nanoparticles, approximately ~10 nm in size. Furthermore, at higher temperatures, TFMAQ-UBD nanoparticles showed increased self-assembly behavior that generated particles that were a few micrometers in size, accompanied by cloud points and a lower critical solution temperature (LCST) behavior. In the ex vivo experiments that used TFMAQ-UBD, an intense emission corresponding to the fluorophore TFMAQ was observed in tumors, following administration of TFMAQ-UBD to tumor-bearing mice. In particular, mice exposed to a local heat showed a more intense emission than mice not exposed to the heat. Despite exhibiting superior properties both in vitro and ex vivo, clinical applications of TFMAQ-UBD is associated with multiple challenges, including low water-solubility and a lengthy synthesis process, which makes it difficult to prepare in large quantities. In particular, in vivo examinations that used aqueous solutions of TFMAQ-UBD, symptoms of thrombosis were often observed in the lungs, in mice. Thus, we considered an alternative to TFMAQ-UBD that uses a TFMAQ derivative to design and prepare TFMAQ-diEg4 (Scheme 1); the resulting compound had a TFMAQ base with two tetraethylene glycol chains at the amino group. Herein, we describe the size distribution and morphology of the self-assembled TFMAQ-diEg4 molecules. Furthermore, the in vitro thermo-responsive properties of TFMAQ-diEg4 are described and an ex vivo study of fluorescence tumor-imaging, using TFMAQ-diEg4, was conducted.

## 2. Results and Discussion

### 2.1. Synthesis

The water-soluble and fluorescent TFMAQ-diEg4 was prepared according to previously reported methods [21,25] and was synthesized as a yellow fluorescent oil with a total yield of 35%. Synthetic route of TFMAQ-diEg4 is shown in Scheme S1. Moreover, ^1^H NMR of TFMAQ-Eg4 and –diEg4 are shown in Figures S1 and S2.

### 2.2. Solubility for Aqueous Solution

The solubility of TFMAQ-diEg4 in aqueous solutions was investigated, as the prevention of thrombosis is of significance for in vivo examinations. The solubility of TFMAQ-UBD, which has been previously reported as fluorescent probes [20], showed a limited solubility of below 20 mM. However, the resulting TFMAQ-diEg4 showed a high solubility of over 20 mM, in aqueous solutions, indicating that it could not only prevent thrombosis but could also allow for various in vivo examinations, such as single intravenous administration and intravenous drips, with or without local heating, around the tumor tissues.

### 2.3. Self-Assembly Behavior

**Figure 1:**
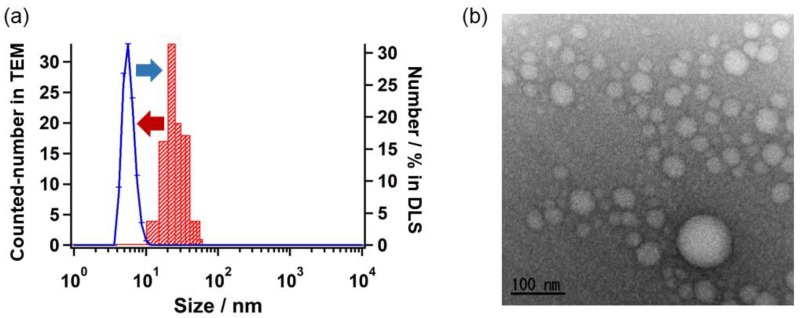
Sizes and morphologies of the particles. (**a**) Plots of the number of nanoparticles in the 5.0 mM TFMAQ-diEg4 solution, obtained using DLS as a function of the *D*_H_ value (right axis; represented as a line graph), and those obtained using TEM as a function of size (left axis; represented as a histogram). (**b**) A representative TEM imaging of the TFMAQ-diEg4 solution (5.0 mM); the scale bar indicates 100 nm.

To investigate the self-assembly behavior of TFMAQ-diEg4 in aqueous solutions, dynamic light scattering was performed at 25 °C, and the hydrodynamic diameter (*D*_H_) of the self-assemblies was determined. TFMAQ-diEg4 solutions of 0.25 mM and 5.0 mM were prepared and used for these experiments. In the 0.25 mM solution, *D*_H_ values were irregular, indicating that the light scattering intensity was insufficient for determining the value of *D*_H_, in the diluted solution. This suggested that in the 0.25 mM solution, soluble TFMAQ-diEg4 exists as small-sized molecules, likely as monomers and dimers. Thus, DLS was performed using the 5.0 mM solution. In the 5.0 mM solution, *D*_H_ was constant, which indicated that a sufficient scattering intensity was obtained. To show the size distribution of the self-assembly, the numbers have been plotted as functions of *D*_H_ value, in Figure 1a, using the right axis as a reference. Notably, a single-peak was observed in the range of 6–8 nm, which suggested the self-assembly of monomers in the 5.0 mM solution. Furthermore, to determine the morphology of the self-assemblies, we conducted transmission electron microscopy (TEM) using the negative stain gadolinium acetate, and observed the spherical nanoparticles in the TEM image (Figure 1b). Size distributions of individual nanoparticle were determined and are plotted in Figure 1a, using the left axis as a reference. As such, the resulting nanoparticles were approximately 20 nm in size, with a relatively poly-dispersed state. The size obtained by the TEM imaging was larger than that obtained by DLS because the nanoparticles collapsed and spread when placed on the TEM copper grid.

### 2.4. Temperature Dependence of Self-Assembly

**Figure 2:**
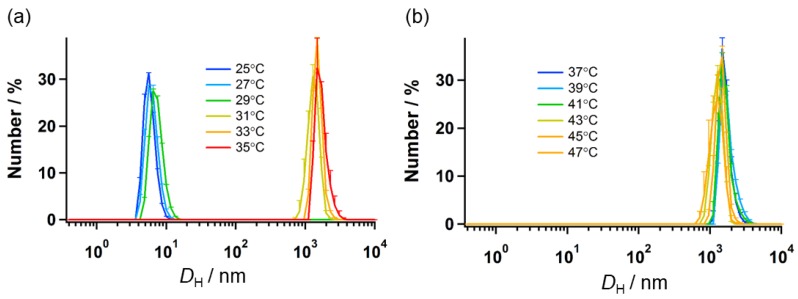
Size variations of the heat-triggered particles. *D*_H_ values of the 5.0 mM TFMAQ-diEg4 solutions were determined using the DLS, at various temperatures. *D*_H_ values are presented between (**a**) 25–35 °C, and (**b**) 37–47 °C.

Temperature-mediated self-assembly occurs in compounds having OEG chains, such as a UBD analog. This behavior is caused by the dehydration process around the OEGs, derived from the cleavage of the hydrogen bonds between OEGs and water molecules [15,16,17,20]. DLS was used to examine the temperature-mediated size variations of the spherical TFMAQ-diEg4 nanoparticles. The size of the nanoparticles was between 6 and 7 nm, at temperatures up to 29 °C, (Figure 2a); however, the size increased up to 2000 nm, at 31 °C (Figure 2a). Furthermore, the particle sizes were maintained above 1000 nm, at temperatures of up to 47 °C, (Figure 2b). These results suggested that in the 5.0 mM solution, the cloud point occurred at approximately 30 °C. 

**Figure 3:**
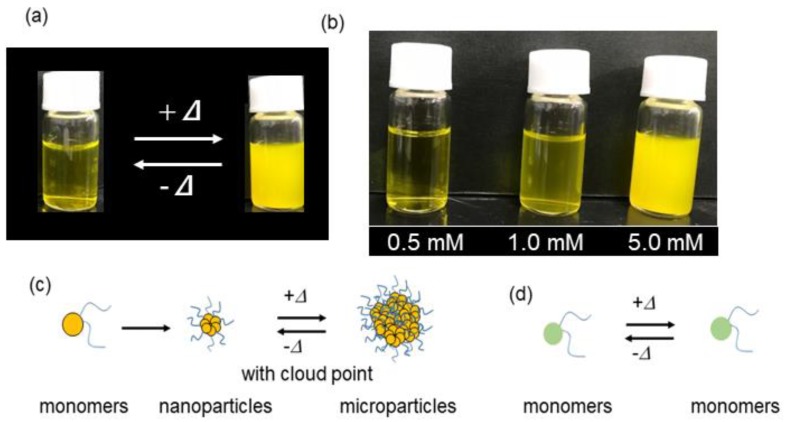
(**a**) Representative image of the vials containing 5.0 mM TFMAQ-diEg4 solutions, at 20 °C (**left**) and 30 °C (**right**). (**b**) Representative image of TFMAQ-diEg4 solutions of various concentrations, at 37 °C. The proposed self-assembly mechanisms are given for the concentrated solutions (**c**) and diluted solution with or without heat (**d**).

Heat-triggered self-assembly was also confirmed visually in vials containing TFMAQ-diEg4 solution (Figure 3a). Solutions of 1.0 and 5.0 mM TFMAQ-diEg4 were transparent at 25 °C, and became cloudy at 37 °C because of the presence of microparticles possessing intense light-scattering properties, which was consistent with the DLS results. In contrast, the 0.5 mM solution was still transparent at 37 °C (Figure 3b). The concentration dependence is explained as follows and is depicted in Figure 3c,d. In solutions of 1.0 mM and higher, the monomers of TFMAQ-diEg4 were allowed to self-assemble into ~20 nm sized-nanoparticles, and then exposed to heat, transformed these nanoparticles to ~2000 nm sized-microparticles (Figure 3c). On the contrary, the 0.5 mM solution produced small-sized molecules, such as monomers, even at high temperatures (Figure 3d).

### 2.5. UV-Vis and Fluorescence Properties

**Figure 4:**
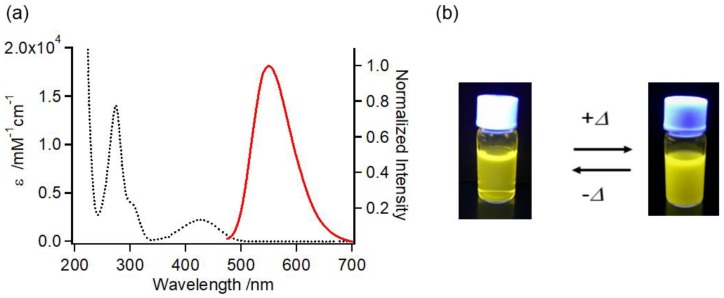
Physical properties of TFMAQ-diEg4. (**a**) The absorption (black dotted line; left axis) and normalized fluorescence (solid red line; right axis) spectra of 20 μM TFMAQ-diEg4, in water. Fluorescence spectrum was obtained using an excitation wavelength of 430 nm. (**b**) Representative image of vials containing 5.0 mM TFMAQ-diEg4 aqueous solution under irradiation, at 365 nm.

To investigate the utility of TFMAQ-diEg4 as a fluorescent bio-imaging probe, absorption and fluorescence spectra of 20 μM water solutions were measured, at 25 °C (Figure 4a). Absorption was observed at a maximum of 428 nm (ε: 1.1 × 10^3^ M^−1^ cm^−1^), which indicated the transition on the basis of the charge transfer of the TFMAQ framework [21,22,23,24]. Emission spectra were observed at a maximum of 547 nm (excitation at 430 nm), and the fluorescence quantum yield was estimated to be 0.07, which was consistent with that of TFMAQ-UBD [20]. Accordingly, TFMAQ-diEg4 possessed sufficient fluorescence intensity to function as a fluorescent bio-imaging probe, similar to TFMAQ-UBD and other chromophores, such as an aminocoumarin derivative [26]. The absorption and fluorescence spectra are shown in Figure 4a, along with the representative images of vials containing aqueous solutions of TFMAQ-diEg4, under irradiation at 365 nm, in Figure 4b. Temperature dependence of fluorescence spectra are shown in Figure S3.

### 2.6. Tumor-Imaging with TFMAQ-diEg4

Fluorescence imaging was performed on tumor-bearing mice administered TFMAQ-diEg4, in various conditions. To reveal correlations between the tumor accumulation and the concentration effects of TFMAQ-diEg4, samples were prepared as 2.0 mM and 0.2 mM saline solutions. From the viewpoint of solubility, a 20 mM solution was invalid for the examination. There were also two groups of mice: in the first group, the tissue surrounding the tumor was heated, whereas, in the second group, local heating was not applied. Mice were administered 200 μL TFMAQ-diEg4 in saline, via the tail vein, whereas in the subset of mice, this treatment was followed by an intravenous injection. To summarize, the five treatments were as follows:

(1) Single-injection of 2.0 mM TFMAQ-diEg4 in saline, via the tail vein, *without* local heat.
(2) Single-injection of 2.0 mM TFMAQ-diEg4 in saline, via the tail vein, *with* local heat.
(3) Single-injection of 2.0 mM TFMAQ-diEg4 in saline, via the tail vein and drip via the tail vein, *with* local heat.
(4) Single-injection of 0.2 mM TFMAQ-diEg4 in saline, via the tail vein and drip, *without* local heat.
(5) Single-injection of 0.2 mM TFMAQ-diEg4 in saline, via the tail vein and drip with local heat.

The protocols for each group are summarized in Table 1 and Figure S4. Given the variation in individual mouse volume and weight, the in vivo TFMAQ-diEg4 concentrations were estimated to be, approximately, 0.5 mM in groups 1 and 2, over 1.0 mM in group 3, and over 0.1 mM in groups 4 and 5. The given concentrated-solutions that were maintained at 23 °C, was injected into the individual mouse. To heat the tumor tissue, hot water (40 °C) was circulated around the tumor tissues, to maintain a temperature of approximately 37 °C, at the tumor site. The accumulation of TFMAQ-diEg4 was determined using the fluorescence intensities of the tumor tissues that were removed from the mice, after euthanasia.

**Figure 5:**
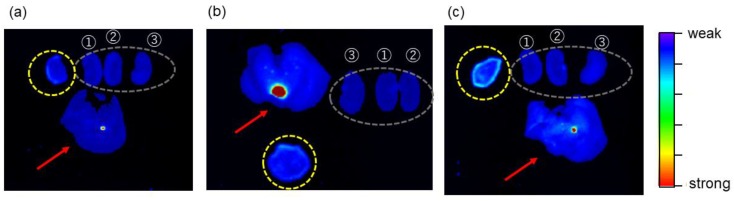
Fluorescence imaging of tumors (broken yellow circles), kidneys (broken gray ovals), and livers (red arrows) was visualized using TFMAQ-diEg4 emission. The numbers 1 to 3 along the kidneys indicate a kidney cut in half (1 and 2) or a whole kidney (3). Representative images shown are of groups 1 (**a**), 2 (**b**), and 3 (**c**).

**Figure 6:**
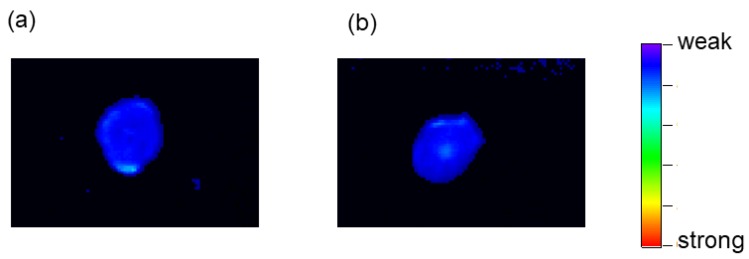
Representative images of tumor tissues visualized using the TFMAQ-diEg4 emission. Images from groups 4 (**a**) and 5 (**b**) are shown.

In Groups 1 and 2, intense TFMAQ-diEg4 emission in the tumor tissue was not observed, which was likely because TFMAQ-diEg4 did not accumulate within this tissue; however, weak emissions, at the edge of the tumor tissues, were observed (Figure 5a,b). Notably, fluorescence intensity observed in tissues from Group 2 was slightly higher than that from group 1. In contrast, high-intensity emissions at the edge and the center of the tumor tissue were observed, clearly, in Group 3 (Figure 5c). Surprisingly, the emission intensities of the tumor tissues in Groups 4 and 5 were very weak, even though these groups received drips of TFMAQ-diEg4 (Figure 6a,b). Furthermore, local heat to the tumor tissue induced a relative increase in emission intensity, as highlighted by comparing emission intensity in Groups 1 and 2, which indicated that TFMAQ-diEg4 accumulation, within the tumor tissues, was triggered by heat. However, the difference in emission intensity between Groups 1 and 2 was small. Given that the approximate in vivo concentration was 0.5 mM and the in vitro data suggested that these concentrations form only small molecules (monomers and dimers), it was likely that the mice metabolized the molecules. Samples from Group 3, however, exhibited high-intensity emissions, within the tumor tissue, which could be clearly observed because of the accumulation of the TFMAQ-diEg4. Thus, the difference between Groups 1, 2, and 3 might be related to the formation of nanoparticles. Within the body of mice in Group 3, nanoparticles formed, as the drip maintained relatively high concentrations of the TFMAQ-diEg4. In contrast, TFMAQ-diEg4 concentration in mice, from Groups 4 and 5, was too low to form nanoparticles, which allowed the metabolism of the molecules, similar to that in Groups 1 and 2.

## 3. Materials and Methods 

### 3.1. Instruments and Sample Preparation

Various spectra using as following instruments. Infrared spectra (420 FT-IR, JASCO Ltd., Tokyo, Japan). UV-Vis spectra (V570 and V760, JASCO Ltd., Tokyo, Japan). Fluorescence spectra (FP8500, JASCO Ltd., Tokyo, Japan). Fluorescence quantum yield (Spectrofluorometer equipped with a calibrated integrating sphere system, JASCO Ltd., Tokyo, Japan). ^1^H and ^13^C NMR (AVANCE III 500, Bruker Biospin Ltd., MA, USA or 500 Fourier transform NMR spectrometer, Agilent Ltd., CA, USA). High-resolution electrospray ionization mass spectra (microTOF mass spectrometer, Bruker Daltonics Ltd., MA, USA).

DLS (Zetasizer Nano ZS, Malvern Instrument Ltd., Worcestershire, UK) equipped with a helium-neon laser (wavelength: 633 nm) at a scattering angle of 173°. The viscosity values at each temperature were investigated and the given values were used for the estimation of the *D*_H_ value. TEM images were obtained using an FE-TEM (JEM-2010F, JEOL Ltd., Tokyo, Japan), operating at 200 kV. Samples for the TEM observations were prepared by first attaching the grid to a collodion membrane (Nisshin EM, Tokyo, Japan) and treating it with a hydrophilic coating for 30 s. (HDT-400, JEOL, Tokyo, Japan). The sample (5 mL) was then mounted on the grid and after 30 s, the residual solution was absorbed using filtrate paper. Diluted EM stainer (5 mL) was then added to the grid, and after 30 s, the residual solution was absorbed using the filtrate paper, and then dried over a blower. The EM stainer was prepared using gadolinium acetate, diluted 1/10 with distilled water, and then filtered using a 0.45-mM filter.

### 3.2. Materials

Unless otherwise stated, the reagents and solvents were used as provided with no further purifications. All reagents and solvents were purchased from Kanto Chemical Co. Inc. (Tokyo, Japan). TFMAQ and 2,5,8,11-tetraoxatridecan-13-yl 4-methylbenzenesulfonate were prepared, according to methods reported in a previous study [21].

Synthesis of 2,4-trifluoromethyl-7-*N*-(2,5,8,11-tetraoxatridecane-13-yl)-aminoquinoline (TFMAQ-Eg4) was done as follows. First, 1.55 g TFMAQ (5.53 mmol) and 1.11 g of 60% NaH (27.6 mmol) were added to a distilled solution of THF in an ice bath. The reaction mixture was stirred for 20 min. Then, 11 mL distilled THF solution containing 1.76 g 2,5,8,11-tetraoxatridecan-13-yl 4-methylbenzenesulfonate (5.53 mmol) was added drop-wise, to the reaction mixture. The reaction was stirred overnight, while the temperature of the reaction was raised to the room temperature. The reaction mixture was then quenched by the addition of MeOH, and then extracted three times, with Et_2_O. The combined organic layer was washed with saturated NH_4_Cl_(aq)_ and dried over MgSO_4_. The MgSO_4_ was then removed by a filter paper. The filtrate was evaporated, in vacuum, and the residue was purified by silica gel column chromatography, using CHCl_3_/AcOEt (30/1-1/1) as eluents. Using this methodology, 1.34 g of TFMAQ-Eg4 (2.85 mmol; 53% yield) was obtained as a yellow fluorescent oil.

TFMAQ-Eg4 was assigned using following spectra; IR (neat on NaCl) 3349 (ν_as_ NH), 2898 (ν_as_ CH_3_), 1632 (ν_s_ C=N), 1455 (ν_s_ C=C or C=N), 1277 (ν_s_ C-F), 1250 (ν_s_ C-F), 1140 (ν_s_ C-F) cm^−1^. ^1^H-NMR (CDCl_3_, 500 MHz) δ 7.93 (*dd*, *J* = 9.3, 1.9 Hz, 1H), 7.65 (*s*, 1H), 7.21 (*dd*, *J* = 9.3, 2.5 Hz, 1H), 7.16 (*d*, *J* = 2.4 Hz, 1H) 5.28 (*s*, 1H), 3.80 (*t*, *J* = 4.9 Hz, 2H), 3.70–3.65 (*m*, 10H), 3.56–3.55 (*m*, 2H), 3.45 (*t*, *J* = 5.1, 2H), 3.37 (*s*, 3H). ^13^C-NMR (CDCl_3_, 126 MHz) δ 151.0, 150.5, 147.6 (*q*, *J* = 35.0 Hz), 135.7 (*q*, *J* = 32.3 Hz), 126.5, 124.5, 123.1 (*q*, *J* = 275.5 Hz), 121.3 (*q*, *J* = 275.7 Hz), 117.1, 109.3, 105.0, 71.9, 70.6, 70.6, 70.6, 70.5, 70.4, 68.8, 59.0, 43.0. HRMS(ESI): *m/z* calcd. for C_20_H_25_F_6_N_2_O_4_ [M + H]^+^: 471.1713 Found: 471.1693. 

Synthesis of 2,4-trifluoromethyl-7-*N*-bis(2,5,8,11-tetraoxatridecane-13-yl)-aminoquinoline (TFMAQ-diEg4) was done as follows. To 10 mL distilled THF, 1.10 g TFMAQ-Eg4 (2.58 mmol) and 515 mg 60% NaH (12.9 mmol) were added, and the reaction mixture was stirred on an ice bath, for 30 min. A solution of 2.46 g 2,5,8,11-tetraoxatridecan-13-yl 4-methylbenzenesulfonate (7.74 mmol) in 10 mL distilled THF was then added drop-wise, and the mixture was stirred overnight, while the temperature was increased to room temperature. The resulting solution was diluted with MeOH and water, and then extracted three times with Et_2_O. The combined organic phase was washed with saturated NH_4_Cl_(aq)_ and dried over MgSO_4_. After removal of MgSO_4_, the filtrate was evaporated and the residue was dried in a vacuum. The residue was chromatographed twice on silica gel with CHCl_3_/MeOH (50/1-10/1), as an eluent, to produce 664 mg TFMAQ-diEg4 (yield 61%), as a fluorescent yellow oil.

TFMAQ-diEg4 was assigned using following spectra; IR (neat on NaCl): 2872 (ν_s_ CH_3_), 1619 (ν_s_ C=N), 1459 (ν_s_ C=C or C=N), 1275 (ν_s_ C-F), 1125 (ν_s_ C-F), 818 (δ_t_ CH) cm^−1^. ^1^H-NMR (CDCl_3_, 500 MHz): δ 7.98 (*dd*, *J* = 9.6, 1.9 Hz, 1H) 7.62 (*s*, 1H) 7.47 (*dd*, *J* = 9.6, 2.8 Hz, 1H) 7.28 (*d*, *J* = 2.7 Hz, 1H) 3.77–3.74 (*m*, 8H) 3.63–3.61 (*m*, 12H) 3.52–3.50 (*m*, 4H) 3.36 (*s*, 6H). HRMS(ESI): *m/z* calcd. for C_29_H_43_F_6_N_2_O_8_ [M + H]^+^: 661.2918 Found: 661.2908.

No cytotoxicity of TFMAQ-diEg4, in vitro or in vivo, was examined.

### 3.3. Temperature Control in Mice with and without Local Heating around the Tumor Tissue

After anesthetization, the rectal temperature of each individual mouse was maintained at 33 °C, using a heating lamp. For local heating around the tumor tissue, the temperature of the surrounding tissue was maintained above 37 °C, using a heating pad with circulating hot water (i.e., tumors contacting the pad were maintained at 40 °C).

### 3.4. Single and Drip Administration via Tail Vein

*BALB*/*c* nude mice bearing colon-26 tumors on the lower back were used. For the single administration, TFMAQ-diEg4 (2 mM, 200 μL) was administered via the mouse coccygeal (tail) vein. After 2.5 h, the mice were euthanized, tumor tissues were removed, and fluorescence intensity was measured. For the drip administration, TFMAQ-diEg4 (2 mM, 200 μL) was administered via the mouse coccygeal (tail) vein, and the TFMAQ-diEg4 (2 mM) was subsequently administered through a drip (100 μL/h), for 2.0 h. At 30 min, after the drip system was stopped, the mice were euthanized. The tumor and other tissues were removed and their fluorescence images were captured.

## 4. Conclusions

For the development of an alternative to TFMAQ-UBD, we synthesized TFMAQ-diEg4, an emissive molecule with an aminoquinoline framework. TFMAQ-diEg4 had advantages of a higher water solubility and a shorter synthesis process than those of TFMAQ-UBD. Taking advantage of the thermo-responsive properties of TFMAQ-diEg4, we showed that TFMAQ-diEg4 was useful as a fluorescent probe for tumor-imaging. In particular, administrations using drip into mice have never been conducted. In the in vitro experiments, high concentrations (over 1.0 mM) of TFMAQ-diEg4 in aqueous solutions showed a self-assembly behavior, which led to the formation of 6–7-nm nanoparticles. When the solutions were exposed to heat, the nanoparticles transformed into ~2000-nm microparticles. A saline solution of TFMAQ-diEg4 was then administered to tumor-bearing mice and when the in vivo concentration of the TFMAQ-diEg4 was higher than 1.0 mM, the particles were accumulated in the tumor tissue and observed as a high fluorescence intensity. In contrast, TFMAQ-diEg4 at below 1.0 mM showed low fluorescence intensity within the tumor tissues. These results indicated that the formation of nanoparticles and the transformation of nanoparticles to microparticles are key to their accumulation within tumor tissues. In addition, during in vivo examinations, we observed no symptoms of thrombosis in mice. Studies on the construction of a fluorescence probe, possessing not only thermo-responsive properties but also emissive properties in the near-red region, are underway, with the aim of developing practical fluorescence imaging tools for tumor imaging. 

## Figures and Tables

**Table 1:** Conditions of the treatment groups and the fluorescence imaging results, following the administration of TFMAQ-diEg4.

Groups *	1	2	3	4	5
Concentration of the vial	2.0 mM	2.0 mM	2.0 mM	0.2 mM	0.2 mM
Concentration in the body ^#^	0.5 mM	0.5 mM	Over 1.0 mM	Over 0.1 mM	Over 0.1 mM
Heat at tumor	No	Yes	Yes	No	Yes
Intravenous (i.v.) administration via the tail vein	Single-i.v. of 200 μL	Single-i.v. of 200 μL	Single-i.v. of 200 μL and then drip of 100 μL/h for 2 h	Single-i.v. of 200 μL and then drip of 100 μL/h for 2 h	Single-i.v. of 200 μL and then drip of 100 μL/h for 2 h
Emission intensity	Weak **	Weak **	Strong	Weak	Weak

* See the text; ** Group 2 showed slightly stronger fluorescence than group 1; ^#^ Estimations were made based on the volume and the weight of the individual mouse.

## Supplementary Materials

The following are available online at http://www.mdpi.com/2079-4991/8/10/782/s1, Scheme S1: Synthetic route of TFMAQ-diEg4. Figure S1: ^1^H NMR spectrum of TFMAQ-diEg4 in CDCl_3_. Arrow indicate ^1^H of waters. Figure S2: ^1^H NMR spectrum of TFMAQ-Eg4 in CDCl_3_. Arrow indicate ^1^H of waters. Figure S3: Temperature dependence of fluorescence spectra of TFMAQ-diEg4. Figure S4. Illustrations of mouse that are controlled the body temperature. (a) and (b) indicate the condition without and with the local heating around tumor tissues.
