# Supplementary material for: Fluorescence Tumor-Imaging Using a Thermo-Responsive Molecule with an Emissive Aminoquinoline Derivative
Source: Nanomaterials (Basel). 2018 Oct 2;8(10):782. doi: 10.3390/nano8100782 (PMC6215166; doi:10.3390/nano8100782)
Supplement: Supplementary file 1 [file nanomaterials-08-00782-s001.pdf]

Supplementary Materials

Fluorescence tumor-imaging using a thermo-responsive molecule with an emissive aminoquinoline derivative

Takeru Araki [1], Yasufumi Fuchi [2], Shuhei Murayama [3,4], Ryoma Shiraishi [1], Tokimi Oyama [2], Mariko Aso [1], Ichio Aoki [4], Shigeki Kobayashi [2], Ken-ichi Yamada [1] and Satoru Karasawa [2,5,*]

[1] Graduate School of Pharmaceutical Sciences, Kyushu University, 3-1-1 Maidashi, Higashi-Ku, Fukuoka 812-8582, Japan; araking417@gmail.com (T.A.); oirawawhitydaze0709@gmail.com (R.S.); aso@phar.kyushu-u.ac.jp (M.A.); kenyamada@phar.kyushu-u.ac.jp (K.Y.)

[2] Faculty of Pharmaceutical Sciences, Showa Pharmaceutical University, Machida, Tokyo 194-8543, Japan; fuchi@ac.shoyaku.ac.jp (Y.F.); b14030@ug.shoyaku.ac.jp (T.O.); kobayasi@ac.shoyaku.ac.jp (S.K.)

[3] Department of Bioanalytical Chemistry, School of Pharmacy, Showa University, 1-5-8 Hatanodai, Shinagawa-ku, Tokyo 142-8555, Japan; s.murayama@pharm.showa-u.ac.jp

[4] Department of Molecular Imaging and Theranostics, National Institute of Radiological Sciences (NIRS), QST, Anagawa 4-9-1, Inage, Chiba 263-8555, Japan; iaoki.jp@gmail.com

[5] PRESTO, Japan Science and Technology Agency, Kawaguchi 332-0012, Japan

* Correspondence: karasawa@ac.shoyaku.ac.jp; Tel.: +81-427-211-553

Table of Contents

1. Synthetic route of **TFMAQ-diEg4**. S3
2. Copies of ^{1}H NMR spectrum of **TFMAQ-diEg4**. S4
3. Copies of ^{1}H NMR spectrum of **TFMAQ-Eg4**. S5
4. Temperature dependence of the fluorescence spectra of **TFMAQ-diEg4**. S6
5. Illustrations showing the process of controlling the body temperature, in mice. S7

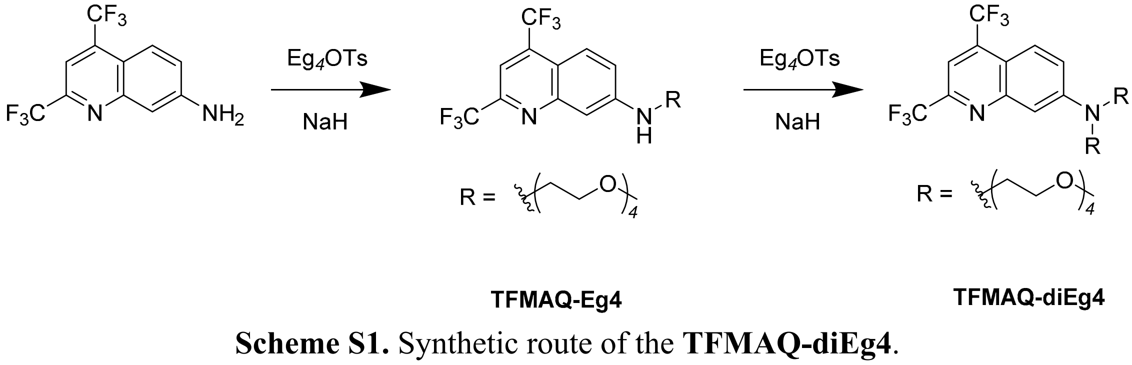

Scheme S1. Synthetic route of the **TFMAQ-diEg4**.

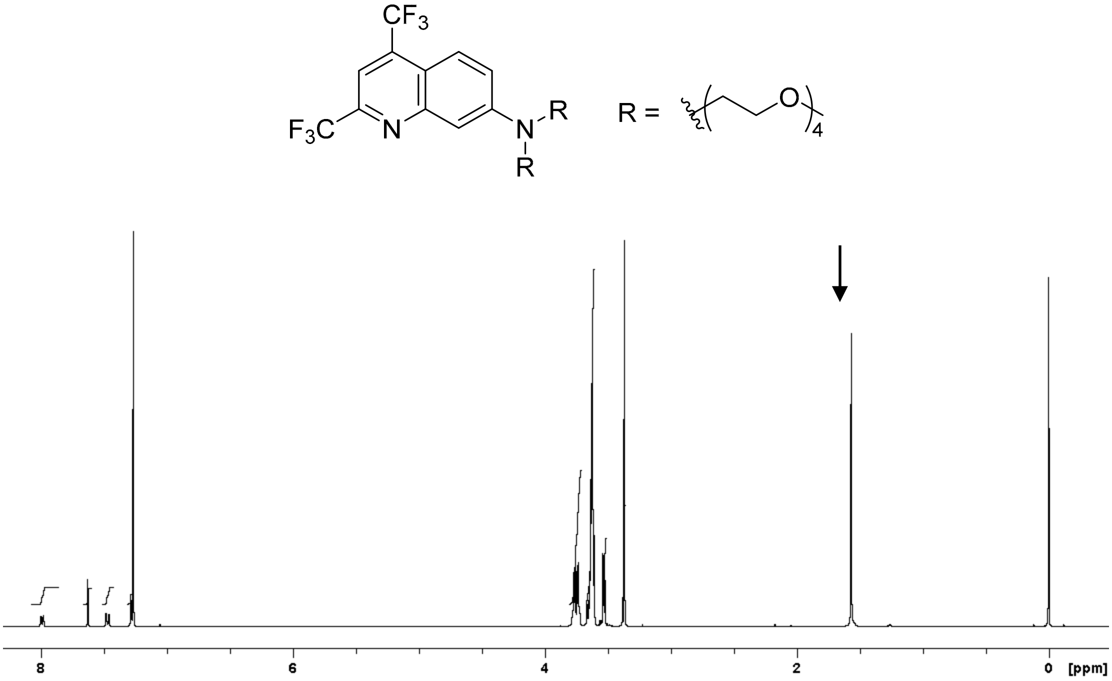

Figure S1. ^{1}H NMR spectrum of **TFMAQ-diEg4** in CDCl3. Arrow indicates ^{1}H of

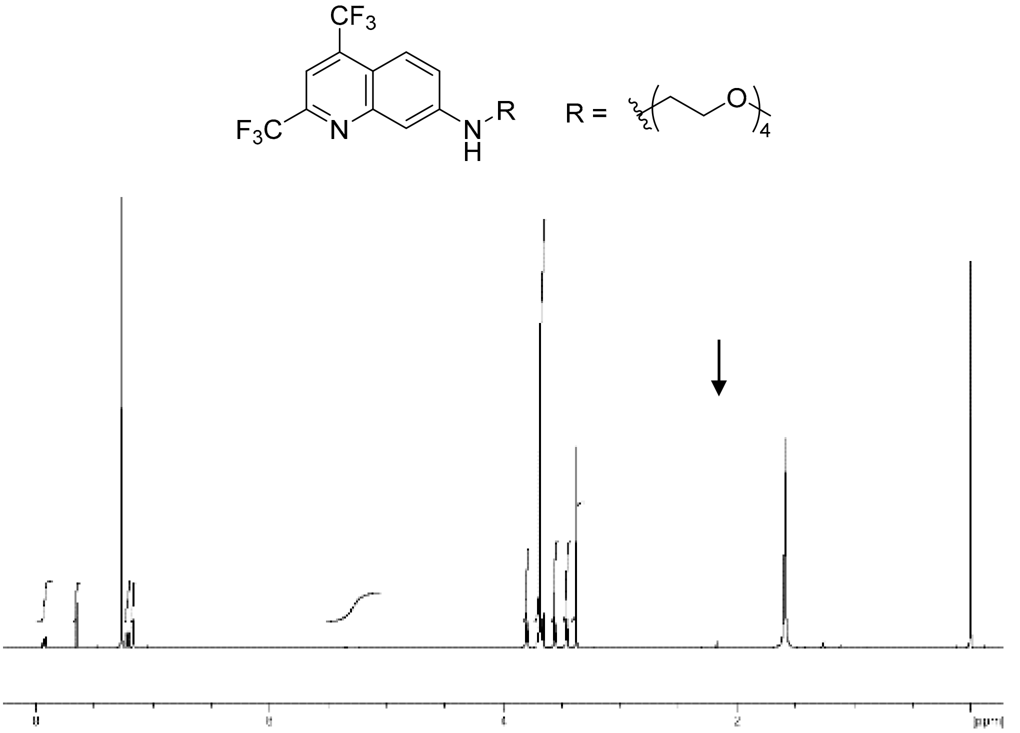

Figure S2. ^{1}H NMR spectrum of **TFMAQ-Eg4** in CDCl3. Arrow indicates ^{1}H of water.

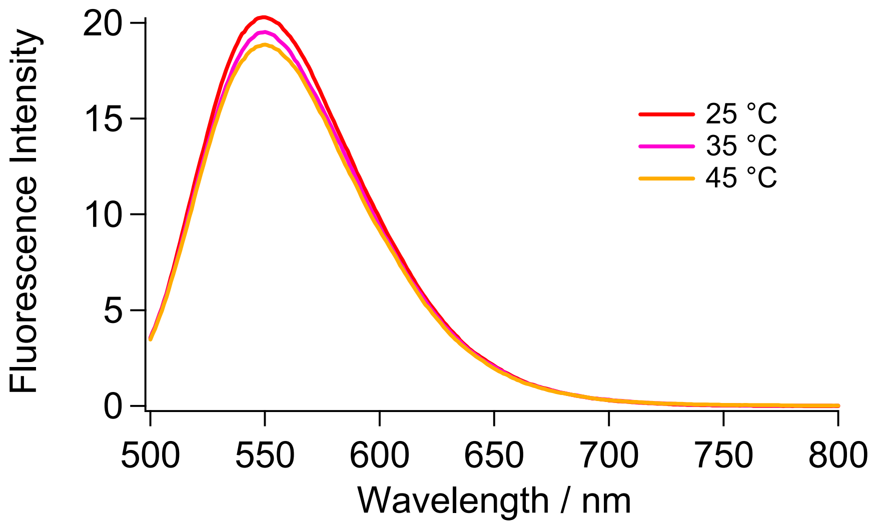

Figure S3. Temperature dependence of the fluorescence spectra of **TFMAQ-diEg4**.

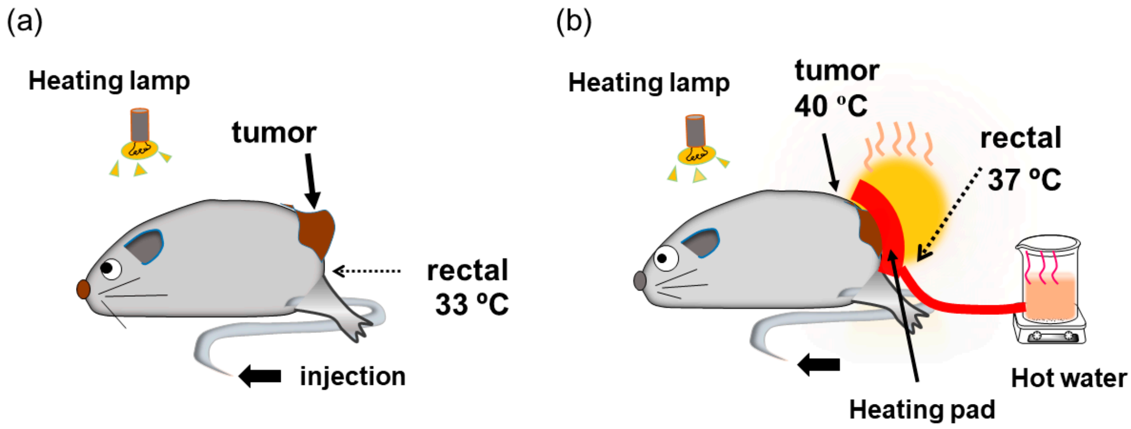

Figure S4. Illustrations showing the process of controlling the body temperature, in mice (a) and (b) indicate the conditions of without and with local heating around tumor tissues, respectively.
